# The emerging importance of immunophilins in fibrosis development

**DOI:** 10.1007/s11010-022-04591-1

**Published:** 2022-10-27

**Authors:** Abdelrahim Alqudah, Rawan AbuDalo, Esam Qnais, Mohammed Wedyan, Muna Oqal, Lana McClements

**Affiliations:** 1 Department of Clinical Pharmacy and Pharmacy Practice, Faculty of Pharmaceutical Sciences, The Hashemite University, Zarqa, Jordan; 2 Department of Biology and Biotechnology, Faculty of Science, The Hashemite University, Zarqa, Jordan; 3 Department of Pharmaceutics and Pharmaceutical Technology, The Hashemite University, Zarqa, Jordan; 4 School of Life Sciences, Faculty of Science, University of Technology Sydney, Sydney, NSW, Australia

**Keywords:** Immunophilins, Fibrosis, Cyclophilin A, FKBP12, Cyclosporin A, FK506

## Abstract

Immunophilins are a family of proteins encompassing FK506-binding proteins (FKBPs) and cyclophilins (Cyps). FKBPs and Cyps exert peptidyl-prolyl *cis-trans* isomerase (PPIase) activity, which facilitates diverse protein folding assembly, or disassembly. In addition, they bind to immunosuppressant medications where FKBPs bind to tacrolimus (FK506) and rapamycin, whereas cyclophilins bind to cyclosporin. Some large immunophilins have domains other than PPIase referred to as tetratricopeptide (TPR) domain, which is involved in heat shock protein 90 (Hsp90) and heat shock protein 70 (Hsp 70) chaperone interaction. The TPR domain confers immunophilins’ pleotropic actions to mediate various physiological and biochemical processes. So far, immunophilins have been implicated to play an important role in pathophysiology of inflammation, cancer and neurodegenerative disorders. However, their importance in the development of fibrosis has not yet been elucidated. In this review we focus on the pivotal functional and mechanistic roles of different immunophilins in fibrosis establishment affecting various organs. The vast majority of the studies reported that cyclophilin A, FKBP12 and FKBP10 likely induce organ fibrosis through the calcineurin or TGF-β pathways. FKBP51 demonstrated a role in myelofibrosis development through calcineurin-dependant pathway, STAT5 or NF-κB pathways. Inhibition of these specific immunophilins has been shown to decrease the extent of fibrosis suggesting that immunophilins could be a novel promising therapeutic target to prevent or reverse fibrosis.

## Introduction

Immunophilins are a family of proteins that include FK506-binding proteins (FKBPs) and cyclophilins (Cyps) [1]. Both FKBPs and Cyps exert peptidyl-prolyl *cis-trans* isomerase (PPIase) activity, which catalyses the isomerization of proline cis-trans peptide bond [2], enhancing diverse protein folding assembly or disassembly. Irregular PPIase activity is associated with the development of cardiovascular disease, atherosclerosis, chronic kidney disease and type II diabetes [1]. In addition, they bind to immunosuppressant medications including tacrolimus (FK506) and rapamycin which bind to FKBPs and cyclosporin that binds to cyclophilins [3].

Notably, some large immunophilins have domains other than PPIase referred to as tetratricopeptide (TPR) domain, with main function involving chaperone interaction with heat shock protein 90 (Hsp90) and heat shock protein 70 (Hsp 70) [4]. These immunophilin-Hsp90/Hsp70 complexes facilitate secondary protein structure folding or unfolding, which is important for cell growth and differentiation [5]. The presence of additional domains confers immunophilins’ pleotropic function once bound to their respective target proteins such as glucocorticoid receptor and nuclear factor κB (NF-κB) to mediate various physiological and biochemical processes, including protein trafficking, receptor signalling, RNA recognition and transcription [6].

Based on their cellular distribution, immunophilins are classified as nuclear (e.g. FKBP25, Cyp33, FKBP13 and FKBPL) [7–9], mitochondrial (e.g. FKBP38, CypD) [10, 11], endoplasmic reticulum (e.g. CypB, FKBP13, FKBP19, FKBP22, FKBP60 and FKBP65) [12], cytoplasmic (e.g. FKBP12, CypA, and FKBPL) [13] and multidomain (e.g. FKBP36, FKBP38, FKBP51, FKBP52, and Cyp40) [14]. FKBP51 and CypA are mitochondrial factors that undergo nuclear-mitochondrial shuttling during stress response to protect cells against oxidative stress [15, 16]. The latter effect was also observed during cells differentiation, where FKBP51and CypA also translocate to the nucleus suggesting that they have a regulatory role in cells differentiation [17, 18]. Moreover, immunophilins are varied in their molecular weight, the higher the molecular weight the more complex the structure that lacks the immunosuppressant effect [19]. Parvulins belong to another group of proteins with PPIase activity outside of the immunophilin family, because they do not bind any specific immunosuppressive drug, hence not affecting protein folding and overall protein function [20]. FKBP like (FKBPL) is a divergent member of the immunophilin family that lacks the PPIase activity despite the presence of the PPI domain, therefore it is unable to bind to immunosuppressant drugs; however, it forms a complex with Hsp90 regulating downstream signalling [6].

In addition to the immunosuppressant effects, immunophilins have shown to have important roles in inflammation [21], cancer [22], cardiovascular disease and neurodegenerative disorders [23–25], by regulating soluble protein retrotransport through the interaction with dynein motors [4], neurodifferentiation and neuroregeneration [26], adipocytes differentiation [27], transcriptional regulation [28], steroid binding capacity [29], cell division [30] and apoptosis [31]. Even though fibrosis develops as a sequela of inflammation, the role of immunophilins in the process of fibrosis development has not yet been elucidated.

Fibrosis is defined as a process of excessive fibrous connective tissue accumulation containing collagen and fibronectin components of extracellular matrix (ECM), known to lead to organ dysfunction and subsequently depending on the organ in question heart failure, kidney disease, end-stage liver disease and idiopathic pulmonary fibrosis [32, 33]. Despite having distinct clinical manifestations, fibrosis is a result of chronic inflammation [34] induced by distinct triggering factors including recurrent exposure to smoke, irritants or toxins, myocardial infarction, obesity, elevated serum cholesterol and poorly controlled hypertension or diabetes [35]. However, regardless of the triggering factors, all fibrosis-associated disorders are characterised by the activation of ECM myofibroblasts towards tissue remodelling following tissue injury or damage [36]. For example, hepatitis C infection leads to myofibroblasts activation increasing collagen accumulation which distorts hepatic architecture, thereby, causing hepatocellular dysfunction and limiting hepatic blood flow causing portal hypertension [37]. On the other hand, left ventricular hypertrophy is associated with fibroblasts differentiation to myofibroblasts with increased synthesis of collagen and fibronectin that leads to ventricular dysfunction [38]. This extensive fibrosis and remodelling can ultimately lead to organ failure and death.

Notably, fibroblasts respond to paracrine signalling from macrophages and lymphocytes, as well as autocrine signalling to migration and differentiation to myofibroblasts [39] and increased secretion of growth factors, cytokines, and metalloproteinases (MMPs), and deposition of ECM proteins, thereby promoting fibrosis [40]. Multiple studies have revealed that TGFβ transcription with subsequent increase in TGFβ protein expression and downstream Smad signalling drives fibroblasts proliferation and differentiation [41] since TGFβ specifically induces α-smooth muscle actin (SMA) expression followed by collagen production [42], suggesting that both TGF-β and α-SMA play a key role in fibrosis pathogenesis. Meanwhile, renin-angiotensin aldosterone system (RAAS), cytokines (TNF-α, IL-21, TGF- β) [43], chemokines (MCP-1), angiogenic factors (VEGF) and caspases also appear to be dysregulated in fibrosis [44]. Therefore, they have been investigated as potential therapeutic targets of anti-fibrotic drugs.

Related to the role of immunophilins, FK506-binding proteins 10 (FKBP 10) was shown to interact with collagen [45], hence playing a crucial role in tissue remodelling [46]. This is suggested to occur through the peptidyl-prolyl *cis-trans* isomerase (PPIase) activity which is needed for proline isomerization facilitating collagen formation and assembly. Previous study has revealed that Fkbp10^−/−^ mouse embryos display a low collagen crosslinking in calvarial collagen [47]. Furthermore, fibrosis due to TGF upregulation appears to be promoted by overexpression of FKBP51 [48]. Thus, this review outlines the key roles and mechanisms that various immunophilins play in fibrosis and discusses their therapeutic target potential towards the development of immunophilin inhibitors that could prevent fibrosis initiation and progression.

## Key immunophilins in lung fibrosis development

Lung fibrosis is a progressive disease leading to scaring and stiffening of the lungs which eventually leads to respiratory failure [49]. In most cases, the diagnosis will be idiopathic pulmonary fibrosis; however, pulmonary fibrosis can be secondary to other causes, such as medications, radiation, environmental pollutants, infections, and genetic susceptibility [50, 51]. Although the pathogenic processes of pulmonary fibrosis are not completely understood [52], several studies have shown immunophilins to play an essential role.

**Fig. 1:**
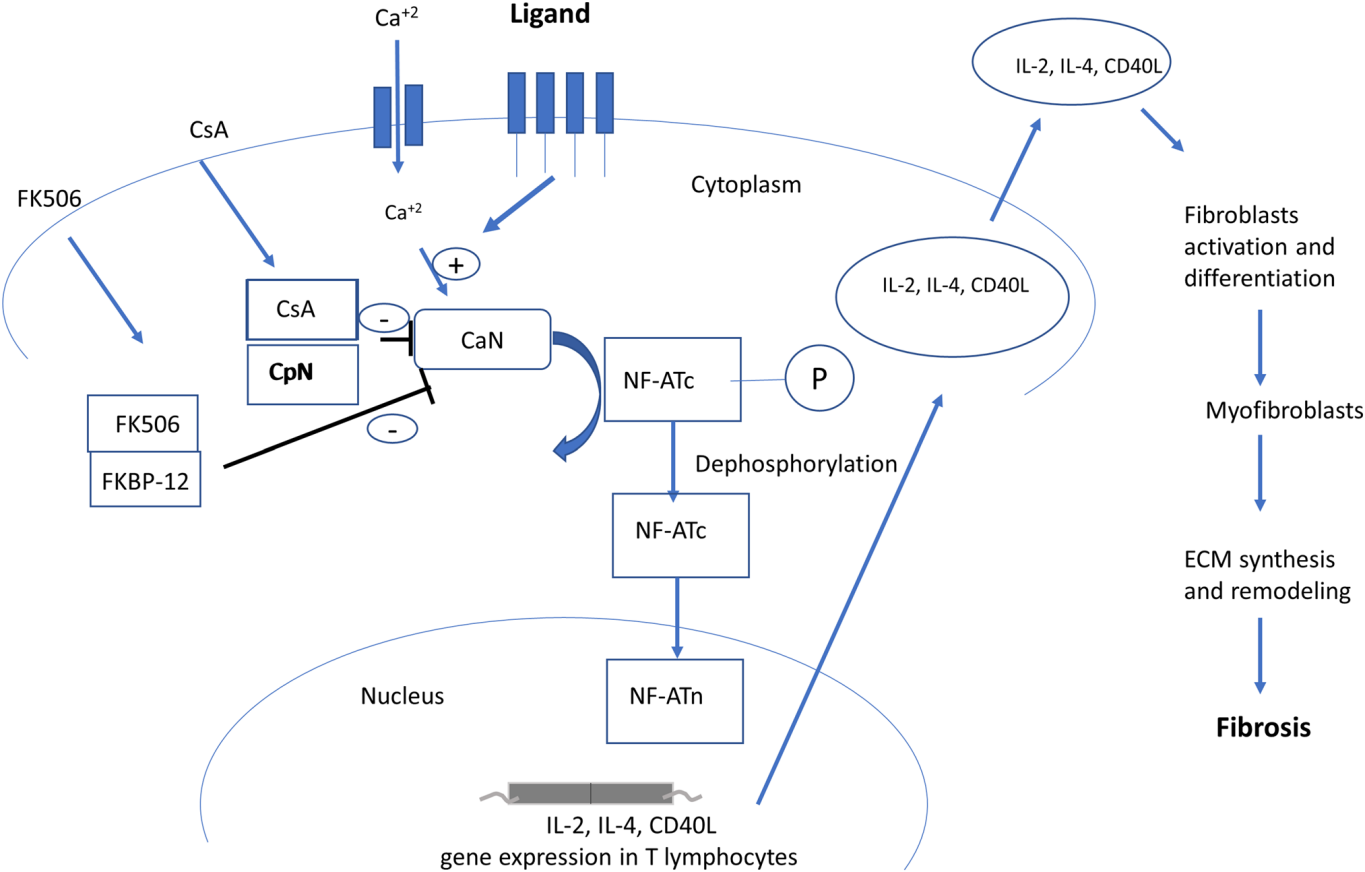
Cyclophilin A and FKBP12 activate T cells which induce fibrosis. Calcineurin (CaN) activity is regulated by Ca + 2/calmodulin. After the engagement of T-cell receptor with its ligand, intracellular Ca + 2 level will interact with calcineurin (CaN) and activates nuclear factor-activated T-cells (NFAT) family members. NFAT then translocate into the nucleus and activate gene expression of cytokines including IL-2, IL-4, and CD40L which then activate fibroblasts differentiation to myofibroblasts which induce extracellular matrix (ECM) synthesis and remodelling to induce fibrosis. Inhibition of CaN pathway with cyclosporin A (CsA) or FK506 will supress fibrosis

Cyclophilins are a family of proteins that facilitate protein folding and play a key role in fibrotic processes including inflammation, activation of apoptotic pathways, and activation of fibroblasts leading to increased collagen secretion [53]. Cyclophilins were found to be highly abundant in fibrotic tissues of the liver and mouth, and inhibition of cyclophilins by cyclosporin was reported to suppress the activity of calcineurin pathway, an important mechanism in fibrosis [54, 55]. Calcineurin (CaN) belongs to a superfamily of protein serine/threonine phosphatases and its activity is regulated by calcium/calmodulin. Following interaction between T-cell receptors with their ligands, calmodulin is activated due to the elevation of and interaction with the intracellular calcium level, activating its phosphatase activity and subsequently nuclear factor-activated T cells (NFAT) family members. NFAT then translocate into the nucleus and activates gene expression of cytokines including IL-2, IL-4, and CD40L which contribute to ECM remodelling, activation of collagen producing fibroblasts, and ultimately fibrosis (Fig. 1) [56]. Thus, inhibition of CaN/NFAT signalling pathway can prevent T-cell activation [57]. Additionally, basic fibroblast growth factor (bFGF) activates several signal factors that stimulate an increase in intracellular calcium levels, essential for cell transition from the G1 phase to the S phase that promote fibroblast proliferation and collagen synthesis with an important role in fibrosis [58, 59]. *Yahong *et al. [60] demonstrated that treating lung fibroblasts with bFGF increased its proliferation by two fold, in addition to 74% and 1.6-fold increase in collagen synthesis and secretion, respectively, which was associated with 60% increase in calcineurin activity. Interestingly, Cyclosporin A (CsA), calcineurin inhibitor, inhibited bFGF-stimulated lung fibroblasts proliferation by 66%, in addition to 37% and 56% inhibition in collagen synthesis and secretion, respectively, which was associated with 44% inhibition of calcineurin activity. Cyclosporin A exerts its effect through the inhibition of cyclophilin A, immunophilin protein member; this step prevents the phosphorylation of NFAT and its translocation into the nucleus therefore inhibiting T-cell activation [61], suggesting that cyclophilin A likely has an important role in lung fibroblasts activation and collagen secretion through CaN/NFAT pathway. Moreover, tacrolimus (FK506) is a calcineurin inhibitor used as an immunosuppressant agent for organ transplant rejection prevention, which exerts its effect through the inhibition of T lymphocytes by forming a complex with FKBP12 [62]. FKBP12 was found to interact with the extracellular domain TGF-beta receptor 1 (TβR-1) which is responsible for the initiation of the downstream signalling [63]. In vitro stimulation of the human lung fibroblasts cell line (TIG-20 cells) with TGF-β significantly increased collagen synthesis; however, treatment with tacrolimus prevented this increase in collagen synthesis. In line with the previous results, tacrolimus reduced the expression of TβR-1 in bleomycin-induced pulmonary fibrosis mice model, which suggests that FKBP12 could induce lung fibrosis through activation of TβR-1 [64]. Furthermore, *Staab-Weijnitz *et al. [46] demonstrated that FK506-binding protein 10 (FKBP10), another member of immunophilin family, is upregulated in lung protein lysates from bleomycin-induced lung fibrosis mouse model that was also confirmed using the microarray analysis of 99 lung samples from idiopathic pulmonary fibrosis patients showing an upregulation of FKBP10 gene expression compared to control, and this upregulation was positively correlated with α-SMA levels, a myofibroblasts marker. Interestingly, FKBP10 knockdown in idiopathic pulmonary fibrosis significantly reduced the expression of collagen I, V, and fibronectin. Since increased fibroblasts migration is a characteristic of idiopathic pulmonary fibrosis, *KnÜppel *et al. [65] studied the effect of FKBP10 deficiency on primary human lung fibroblast cell migration and adhesion. Following exposure to TGF-β1, the results showed that FKBP10 knockdown abrogated primary human lung fibroblast cell migration and adhesion, due to the reduction of collagen VI biosynthesis.

FKBP13, another member of immunophilin protein family, was reported to be highly expressed in lung biopsy samples from patients with idiopathic pulmonary fibrosis compared to control, which was also positively correlated with patient-reported dyspnoea scores. In addition, FKBP13 was positively correlated with α-SMA and unfolded protein response markers, GRP78 and total XBP1, expression, suggesting that higher levels of FKBP13 are associated with increased clinical severity and idiopathic pulmonary fibrosis pathogenicity [66]. In contrary, the same study revealed that FKBP13 knockout mice had a higher sensitivity to damaging effects of bleomycin through contributing to increased inflammatory cells infiltration, lung elastance and fibrosis and impaired resolution of fibrosis, therefore suggesting that FKBP13 might have a protective role against bleomycin-induced lung damage.

Taken together, cyclophilin A, FKBP12, FKBP10, and, FKBP13 have shown an important role in the pathogenesis of lung fibrosis; however, further research should be conducted to strengthen their therapeutic target potential in lung fibrosis treatment.

## Immunophilins and liver fibrosis

Hepatic injury due to hepatitis B (HBV), hepatitis C (HCV), non-alcoholic fatty liver disease (NAFLD) and non-alcoholic steatohepatitis (NASH), the severe form of NAFLD, often leads to hepatic fibrosis and subsequently advanced liver disease [67]. Persistent inflammation and activation of hepatic stellate cells (HSCs) are some of the main characteristics of hepatic fibrosis that lead to tissue remodelling and repair through accumulation of collagen. Progression of liver fibrosis to liver cirrhosis is associated with poor survival and hepatocellular carcinoma development [68]. Therefore, understanding the mechanisms underpinning the development of liver fibrosis is key and can lead to identification of new therapeutic targets.

**Fig. 2:**
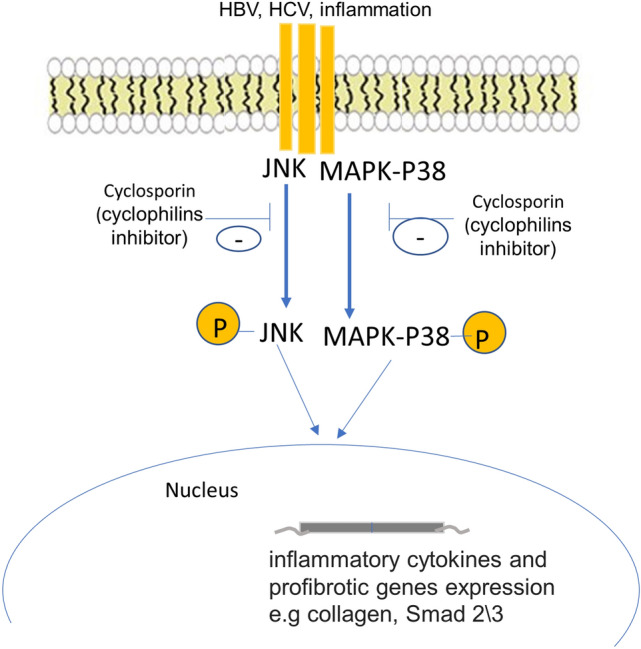
Cyclophilin A activates JNK and MAPK-P38 which induce liver fibrosis. Cyclophilin A activates phosphorylation of *c-jun* N-terminal kinase (JNK) and p38 mitogen-activated protein kinases (MAPKs) which induce hepatic stellate cells (HSCs) growth and collagen production that induce liver fibrosis. Cyclosporin A inhibited cell growth and collagen production which was associated with suppressed phosphorylation of *c-jun* N-terminal kinase (JNK) and p38 mitogen-activated protein kinases (MAPKs)

*Nakamuta *et al. 2005 [69] studied the effect of cyclosporin A, a cyclophilins inhibitor, on HSCs growth and collagen production. In this study, cyclosporin A inhibited cell growth and collagen production through inhibition of *c-jun* N-terminal kinase (JNK) and p38 mitogen-activated protein kinases (MAPKs) phosphorylation (Fig. 2). Similarly, NIM881, cyclosporin A analogue, reduced HSCs growth, collagen production, in addition to increasing collagenase activity and phosphorylation of JNK and p38 [70]. In another study, a novel cyclophilin A inhibitor, NV556, was evaluated for its anti-fibrotic properties using two animal models, Methionine–Cholin-Deficient (MCD) diet model, and STAM model of Nonalcoholic Steatohepatitis model, as well as 3D human liver scaffold in vitro model. NV556 significantly reduced collagen deposition measured by percentage of Sirius red-positive area in the liver in the two animal models and reduced collagen IV expression in addition to significant reduction in LOX gene expression, a marker of activated hepatic stellate cells in vitro [53]. Thus, cyclophilin A inhibitors show promise as future treatments for liver fibrosis through inhibition of the JNK and p38 pathways. Moreover, it is well-established that human immunodeficiency virus-1 (HIV-1) co-infection with hepatitis C virus (HCV) increases the risk of liver fibrosis development [71]. In order to assess the effect of another cyclophilin A inhibitor, CPI-431-32, *Gallay *et al. developed a novel in vitro HCV and HIV-1 co-infection model, including human hepatocytes and CD4 + T lymphocytes. The results of this study demonstrated that CPI-431-32 is capable of inhibiting the replication of both HCV and HIV-1 and their variants [72]. Which suggests that cyclophilin A inhibition during HCV infection could prevent viral replication and ultimately liver fibrosis. Another important mechanism implicated in HCV infection which involves FKBP38, another member of immunophilins. FKBP38 binds to and inhibits mammalian target of rapamycin (mTOR), and consequently, inactivated mTOR fails to phosphorylate downstream targets S6K1 and 4EBP1, which promote cell apoptosis. When cells are infected with HCV, it competes with mTOR for interacting with FKBP38, resulting in the dissociation of mTOR from FKBP38. Thus, activated mTOR phosphorylates downstream targets S6K1 and 4EBP1, which suppress cell apoptosis [73]. Therefore, FKBP38 is essential for HCV persistence and the development of HCV-induced liver fibrosis [74].

## Important roles of immunophilins in cardiac fibrosis and heart disease

Cardiac fibrosis is integral component of many different forms of heart disease. Since the regenerative capacity of the mammalian myocardium is limited, sudden loss of a large number of cardiomyocytes initiates an inflammatory response that replaces the dead myocardium with collagen-based scar [75, 76]. Although, a number of different pathophysiologic conditions can induce cardiac fibrosis including myocardial infarction, ageing, pressure overload, volume overload, hypertrophic cardiomyopathy, diabetes, obesity and toxic insults, the cellular pathogenic mechanism are often similar [77–80].

As discussed above, calcineurin is a calcium-dependent phosphatase that dephosphorylates NFATs which then translocates to the nucleus and activates inflammatory response genes. NFATs are expressed highly in T cells and skeletal muscles, whereas NFAT3 is expressed in different tissues, including the heart. Activation of calcineurin dephosphorylates NFAT3 inducing nuclear expression of cytokines that activate T cells (Fig. 1) [81]. *Molkentin *et al. demonstrated that calcineurin transgenic mice were highly susceptible to sudden death partially due to fibrosis of the ventricular wall. Similar results were demonstrated in NFAT3 transgenic mice showing an extensive fibrosis in the cardiac ventricular wall. Interestingly, as with liver and lung fibrosis, a cyclophilin A inhibitor prevented cardiac fibrosis which was demonstrated in calcineurin transgenic mice [82]. Another immunophilin inhibitor, FK506/FKBP12 inhibitor, was capable of attenuating angiotensin II (Ang II)-induced increase in ERK1/2 and p38 MAPK phosphorylation using rat cardiac fibroblasts. In addition, Ang II-induced rat cardiac fibroblasts proliferation in conjunction with upregulation of fibronectin, pro-collagen, inducible nitric oxide (iNOS) and inflammatory cytokines were inhibited by both cyclophilin A and FK506 inhibitors, suggesting that these inhibitors could attenuate cardiac fibrosis trough inhibition of the calcineurin pathway [83]. In line with the in vitro results in the previous study, inhibition of cyclophilin A and FKBP12 reduced the extent of cardiac fibrosis by inhibition of calcineurin activation in load-induced cardiac hypertrophy rat model, providing further evidence for an important role of cyclophilin A and FKBP12 in inducing cardiac fibrosis through calcineurin pathway [84, 85]. Consistent with the previous reports, cyclophilin A expression is increased in a mouse model of troponin I-induced autoimmune myocarditis, associated with severe cardiac fibrosis, and the inhibition of cyclophilin A with MM284 markedly reduced cardiac fibrosis. When monocytes migration and adhesion was stimulated with recombinant cyclophilin A, there was a significant increase in TNF-α, IL-6, and MMP-9 expression, which was abrogated following addition of cyclophilin A inhibitor, MM284, treatment [86]. *Satoh *et al. showed a marked decreased in collagen content in the perivascular area in apolipoprotein E and cyclophilin A knockout mice model (Apoe^−/−^ Ppia^−/−^) after angiotensin II treatment. These results were evaluated also by isolating cardiac fibroblasts from Apoe^−/−^ and Apoe^−/−^ Ppia^−/−^ mice and determining fibroblasts proliferation and production of reactive oxygen species (ROS) after Ang II treatment. Whilst, cardiac fibroblast proliferation and ROS production were increased from Apoe^−/−^ mice, a dramatic reduction in ROS production was observed in Apoe^−/−^ Ppia^−/−^ mice with no change in proliferation. Additionally, the growth rate of cardiac fibroblasts was higher in Apoe^−/−^ compared to Apoe^−/−^ Ppia^−/−^ fibroblasts and treating cardiac fibroblasts with recombinant cyclophilin A increased cardiac fibroblasts proliferation and migration, which suggest that cyclophilin A contributes to cardiac fibrosis [87]. In a rat-reduced uterine perfusion pressure model of preeclampsia, cardiovascular disorder of pregnancy, cardiac fibrosis was observed measured by the extent of collagen deposition in the heart, and this was in association with a significant increase in cardiac mRNA and protein expression of FK506-binding protein like (FKBPL), a divergent member of the immunophilin family and a key angiogenesis-related protein [24, 88, 89]. Aligned work also demonstrated an increase in FKBPL expression in human cardiac fibroblast cell line exposed to fibrotic stimuli, TGF-β [90]. In a separate study using human plasma samples, high systemic FKBPL levels were reported in people with cardiovascular disease including diastolic dysfunction and established preeclampsia [89, 91]. Whilst the role of FKBPL in cardiac fibrosis is still not fully elucidated, future research should address its potential as both therapeutic target and a biomarker of cardiac fibrosis to enable early diagnosis and prevention of subsequent heart disease.

## FKBP51 importance in myelofibrosis development

Idiopathic myelofibrosis is a myeloproliferative disease characterised by clonal stem cell dysfunction that leads to megakaryocyte hyperplasia and fibrotic cytokines release within the bone marrow environment [92]. Ineffective haematopoiesis leading to pancytopenia and extramedullary haematopoiesis are the main symptoms for this disease due to the collagen deposition in bone marrow tissue [93].

FKBP51 is a member of immunophilin family that can regulate FK506-induced calcineurin inhibition and it was found to be overexpressed in megakaryocytes derived from idiopathic myelofibrosis patients compared to normal megakaryocytes [94]. Furthermore, overexpression of FKBP51 in human megakaryoblastic leukaemia cells, UT-7/mpl, markedly inhibited calcineurin activity, which was associated with induced resistance to apoptosis mediated by cytokines deprivation, suggesting that FKBP51 could be responsible for megakaryocytes hyperplasia through calcineurin-dependant pathway. Moreover, Komura et al. demonstrated that in FKBP51-overexpresssing cell line, STAT5 was sustainably activated in association with JAK2 phosphorylation implying the importance of this mechanism for spontaneous growth of megakaryocytes in idiopathic myelofibrosis In addition, in 2005, Komura et al. also showed that FKBP51 overexpression in UT-7/mpl cell line induced a sustained activation for the nuclear factor κB (NF-κB) after cytokine deprivation and this activation of NF-κB was also detected in proliferating megakaryocytes and in circulating CD34 + patient cells [48]. Interestingly, the inhibition of NF-κB activity did not alter the apoptotic resistance of UT-7/mpl cells and CD34 + megakaryocytes derived from idiopathic myelofibrosis patients, but, inhibited TGF-β1 secretion, highlighting the importance of NF-κB activation in the development of fibrosis in this disease. Taken together, FKBP51 overexpression in idiopathic myelofibrosis disease cells could play an important role in the pathogenesis of this disease.

## Future implications

Overall, a number of immunophilins including cyclophilin A, FKBP12, FKBP38, FKBP51 and FKBPL have shown emerging roles as important pathogenic mechanisms in the development of fibrosis in different organs. However, further research is needed to fully elucidate the therapeutic target or biomarker potential of these immunophilins in lung, liver, myelofibrosis and cardiac fibrosis towards clinical translation and development of much needed anti-fibrotic agents. Furthermore, the importance of immunophilins in fibrosis of other organ including kidneys and gastrointestinal tract should be explored. Although, the vast majority of the studies assessed inhibitors of the conventional immunophilin-based immunosuppressants, cyclosporin A and tacrolimus, these therapeutic strategies are hindered by a number of dangerous side effects related to the immune system. Tacrolimus was able to activate TGF-β signalling in endothelial cells which caused renal arteriolar hyalinosis in renal transplant patients [96]. In addition, immunophilin-based immunosuppressants can cause chronic allograft vasculopathy associated with endothelial oxidative stress, apoptosis and dysfunction that affect the half-life-engrafted solid organ negatively [97]. Moreover, tacrolimus and cyclosporin are able to induce toll-like receptor-4 (TLR4) and the downstream NF-κB that lead to the activation of endothelial cells and increase the production of pro-inflammatory mediators [98]. Therefore, the development of novel therapeutic agents that target other immunophilins, particularly FKBPs, potentially with better side effect profile could be a more viable approach to preventing or reversing fibrosis.

## Summary and conclusion

In this review, we discussed the importance of various immunophilin proteins in organ fibrosis and their downstream signalling that could contribute to the pathogenesis of fibrosis, Table 1. Given this is an area that is still poorly understood with limited research conducted so far, the focus has been on the processes of lung, liver, cardiac fibrosis and myelofibrosis in bone marrow development, which is an area of unmet clinical need with ineffective therapeutic options. The vast majority of scientific reports investigated the importance of targeting cyclophilin A in lung, liver and cardiac fibrosis, showing that this therapeutic strategy can reduce organ fibrosis through inhibition of the CaN/NFAT pathway. FKBP12 appears to have some contribution to the pathogenesis of lung and cardiac fibrosis through the TGF-beta and calcineurin pathway. Similarly, a few studies showed that FKBP10 is implicated in inducing lung fibrosis through the activation of the TGF-beta pathway, whereas FKBP13 showed a conflicting role in lung fibrosis. FKBPL is emerging as a potential biomarker and therapeutic target of cardiac fibrosis; however, this needs to be elucidated further. FKBP51 demonstrated a role in myelofibrosis development through calcineurin-dependent pathway, STAT5 or NF-κB pathways. In conclusion, some members of immunophilin protein family have shown a promising role in organ fibrosis development; however, this is an under-research area that needs further evidence in order to progress immunophilin-based therapeutic or biomarker strategies towards clinical utilisation.

**Table 1:** Summary of immunophilins role in organ fibrosis

Immunophilin	Tested organ for fibrosis	Model used	Mechanism of action	Effect on fibrosis
Cyclophilin A	Lung	Lung fibroblasts	CaN/NFAT pathway	Cyclophilin A inhibition decrease lung fibroblasts proliferation and collagen synthesis and secretion [60]
	Liver	Hepatic stellate cells Methionine–Cholin-Deficient (MCD) diet model STAM model of Nonalcoholic Steatohepatitis model 3D human liver scaffolds	JNK and MAPK	Cyclophilin A inhibition decreased cell growth and collagen production in vitro and decrease collagen deposition in vivo [53, 69, 70]
	Heart	Calcineurin transgenic mice NFAT3 transgenic mice Rat cardiac fibroblasts induced by angiotensin II Load-induced cardiac hypertrophy rat model Troponin I-induced autoimmune myocarditis mouse model Apolipoprotein E and cyclophilin A knockout mice model (Apoe ^−/−^ Ppia^−/−^)	Calcineurin pathway	Cyclophilin inhibition decreased collagen deposition [82] Inhibition of cyclophilin A inhibited rat cardiac fibroblasts proliferation [83] Inhibition of cyclophilin A reduced the extent of cardiac fibrosis [85–87]
FKBP12	Lung	Human lung fibroblasts (TIG-20) Bleomycin-induced pulmonary fibrosis mice	Activation of TβR-1	Inhibition of FKBP12 decrease collagen synthesis [64]
	Heart	Rat cardiac fibroblasts Load-induced cardiac hypertrophy model	Calcineurin pathway	Inhibition of FKBP12 inhibited rat cardiac fibroblasts proliferation [83] Inhibition of FKBP12 reduced the extent of cardiac fibrosis [84]
FKBP10	Lung	Bleomycin-induced pulmonary fibrosis mice Primary human lung fibroblasts	TGF-β pathway	FKBP10 knockdown reduced collagen expression [46] FKBP10 knockdown decreased fibroblasts migration and adhesion [65]
FKBP13	Lung	Lung biopsy from patients with idiopathic pulmonary fibrosis Bleomycin-induced pulmonary fibrosis mice	–	FKBP13 was highly expressed in lung biopsies form patients with idiopathic pulmonary fibrosis FKBP13 downregulation increased fibrosis [66]
FKBPL	Heart	Rat-reduced uterine perfusion pressure model of preeclampsia Human cardiac fibroblast cell line	Not elucidated yet	Cardiac FKBPL expression was increased [24] FKBPL expression was increased in human cardiac fibroblasts stimulated by TGF-β [90]
FKBP51	Myelofibrosis	UT-7/mpl, a human megakaryoblastic leukaemia cells Circulating CD34 + patient cells	Calcineurin-dependant pathway STAT5 NF-κB	FKBP51 overexpression induced megakaryocytes hyperplasia and fibrosis [48, 94, 95]

## Data Availability

Not applicable.
